# Fracture properties of porcine versus human thoracic aortas from tricuspid/bicuspid aortic valve patients via symmetry-constraint Compact Tension testing

**DOI:** 10.1038/s41598-024-83233-6

**Published:** 2025-01-03

**Authors:** Marta Alloisio, Antti Siika, David Freiholtz, Anders Franco-Cereceda, Joy Roy, Hanna M. Björck, T. Christian Gasser

**Affiliations:** 1 Department of Engineering Mechanics, KTH Royal Institute of Technology, Stockholm, Sweden; 2 Department of Molecular Medicine and Surgery, Karolinska Institutet, Stockholm, Sweden; 3 Section of Cardiothoracic Surgery, Department of Molecular Medicine and Surgery, Karolinska Institutet, Stockholm, Sweden; 4 Division of Cardiology, Center for Molecular Medicine, Department of Medicine, Karolinska University Hospital, Karolinska Institutet, Solna, Stockholm, Sweden; 5 Department of Vascular Surgery, Karolinska University Hospital, Karolinska Institutet, Stockholm, Sweden

**Keywords:** Biomedical engineering, Aneurysm

## Abstract

Aneurysm rupture is a life-threatening event, yet its underlying mechanisms remain largely unclear. This study investigated the fracture properties of the thoracic aneurysmatic aorta (TAA) using the symmetry-constraint Compact Tension (symconCT) test and compared results to native and enzymatic-treated porcine aortas’ tests. With age, the aortic stiffness increased, and tissues ruptured at lower fracture energy $D$. Patients with bicuspid aortic valves were more sensitive to age, had stronger aortas and required more $D$ than tricuspid valves individuals (peak load: axial loading 4.42 $\pm$ 1.56 N vs 2.51 $\pm$ 1.60 N; circumferential loading 5.76 $\pm$ 2.43 N vs 4.82 $\pm$ 1.49 N. Fracture energy: axial loading 1.92 $\pm$ 0.60 kJ m^-2^ vs 0.74 $\pm$ 0.50 kJ m^-2^; circumferential loading 2.12 $\pm$ 2.39 kJ m^-2^ vs 1.47 $\pm$ 0.91 kJ m^-2^). Collagen content partly explained the variability in $D$, especially in bicuspid cases. Besides the primary crack, TAAs and enzymatic-treated porcine aortas displayed diffuse and shear-dominated dissection and tearing. As human tissue tests resembled enzymatic-treated porcine aortas, microstructural degeneration, including elastin loss and collagen degeneration, seems to be the main cause of TAA wall weakening. Additionally, a tortuous crack developing during the symconCT test reflected intact fracture toughening mechanisms and might characterize a healthier aorta.

## Introduction

There is a fundamental lack of knowledge concerning the microstructural failure mechanism in soft biological tissue and how this determines its macroscopic fracture/failure behavior^1^. For the most part, this is a consequence of the intricate structural arrangement and the interaction of the tissue’s extracellular matrix constituents, which gives rise to highly complex mechanical behavior and makes the discernment of mechanical processes at supraphysiological loading particularly challenging. Besides vascular tissue fracture being a pivotal factor in triggering clinical events, such as ischemic stroke, myocardial infarction, and aortic dissection or rupture^2^, failure at the microstructural level has been suspected to play an important role in the progression of vascular diseases, such as carotid plaques^3 ^and abdominal aortic aneurysms^4^. The homeostasis between tissue damage and healing that determines the normal blood vessel wall, is likely disrupted in diseased vessels. Healing can then not repair all tissue defects; the disease progresses and eventually results in clinical events^5^. Knowledge of how vascular tissue fracture forms and then propagates might, therefore, enhance patient treatment strategies^6^.

Aortic aneurysm (AA) is a silent cardiovascular disease, characterized by a dilated aorta that may eventually dissect or rupture^7,8^. AA is often associated with factors, including smoking, ageing, genetic disorders and hypertension, all of which may lead to cellular changes, elastic fiber degradation, collagen deposition, and inflammation^9^. Bicuspid aortic valve (BAV), the most common congenital heart malformation, is the most significant risk factor for thoracic AA (TAA)^10^. The presence of a BAV in a non-dilated, normal, aortic setting also has consequences on the structural integrity of the aortic wall. Specifically, an increased collagen turnover and impaired collagen cross-linking have been reported previously^11^. Aneurysmatic BAV patients display a higher strength and stiffness than TAV cases, a factor that may contribute to the prevalence of TAAs and eight-fold higher risk of acute aortic events^12,13^.

Given the different spatial concentrations and orientations of the structural proteins and cell types, the arterial wall is structured into three functionally distinct layers: the intima, the media, and the adventitia^14,15^. The innermost layer, the intima, consists mainly of endothelial cells, forms the interface between blood and the arterial wall, and directs the vasculature’s response to alterations in blood flow. The media comprises a network of smooth muscle cells, collagen fibers and elastin lamellae, whereas the adventitia represents a fibrous layer rich in collagen and fibroblasts. The adventitia shields the media and intima layers from mechanical overload and anchors the vessel to its surroundings^16^.

Elastin and collagen are the most abundant and main load-bearing structural proteins of the aortic extracellular matrix, and together, they ensure the vessel wall’s structural integrity and dominate its passive biomechanical properties^14,15^. Elastin forms the central core of elastic fibers, providing elasticity and resilience to blood vessels. Specifically, elastin determines the mechanical properties of the vessel wall at low strains, and it is fundamental to support recoil in response to high-pressure pulsatility^17^. For this reason, the thickness and the elastin content within the aorta decrease from the side closest to the heart toward the distal sections^16,17^. Pathologies, including aneurysms, result in functional and structural alterations of said proteins, and the related microstructure rearrangements result in a loss of aortic wall integrity and strength^1,6,15^.

Collagen fibers primarily influence macroscopic mechanical properties at higher loading. They contribute stiffness, strength, and toughness^18 ^to the vessel wall, even though only less than 10% are mechanically engaged at mean arterial pressure^19^. In unloaded conditions, the collagen fibers in the aortic wall are undulated and crimped. When the tissue deforms, fibers unfurl, and as they straighten, they recruit to load bearing and give rise to the nonlinear mechanical behavior of the vessel wall^20^. The amount of collagen along the aorta changes, leading to a stiffer collagen-rich abdominal aorta as compared to the collagen-poor thoracic aorta^21^. Apart from the collagen content in the wall, its spatial orientation and the spread in orientation influence the macroscopic mechanical properties^22^. The orientation of the collagen fibers also changes gradually from the highly circumferential alignment in the media towards the more isotropic organization in the adventitia^23^. In addition, collagen contributes to the intrinsic toughening processes of soft biological tissue, as reported in studies on skin^24,25^. Crack tip blunting is accompanied by fibril straightening, fibril reorientation towards the tensile direction, elastic stretching and interfibrillar sliding during crack propagation.

In-vitro mechanical tissue characterization allows for a controlled investigation of the mechanical response of the vessel wall at physiological and supraphysiological stress levels^26^. The acquired data may be used to design and validate constitutive models towards, for example, the study of disease-related effects on the vessel wall properties. Traditional tensile tests (uniaxial^28,29^, biaxial^30,31^, bulge inflation^32,33^) were used to identify the vessel wall’s nonlinear, rate-dependent and anisotropic properties. However, such protocols cannot uncover the tissue’s fracture properties, as they lack stable and controlled fracture propagation. It is also challenging to distinguish between viscoelastic-related and damage-related dissipation.

Numerous experimental protocols direct towards exploring vessel wall fracture properties. The single-notched tensile test localizes the crack advancement for mode I fracture but may still result in unstable crack propagation^34^. The guillotine test controls mode I crack propagation^35 ^but prevents natural rupture mechanisms, such as the pull-out of collagen fibers. The dissection^27,36 ^and the tear propagation^37 ^tests explore dissection failure under (constrained) delamination kinematics. Shear fracture (mode II) has been considered the dominating mechanism for the dissection of arteries and characterized by a shear fracture ring test^27^, for example.

In the present work, we utilize the symmetry-constraint Compact Tension (symconCT) test^38^, a novel experimental protocol to rupture the vascular wall. Here, we studied the fracture properties of the diseased human thoracic aorta collected from TAA patients, and the results were then compared to the porcine (native, collagenase- and elastase-treated) aorta. Histological inspection of the fractured test specimens completed our investigation.

## Results

The symconCT fracture test facilitated stable crack propagation, enabling a thorough investigation of peak load, normalized clamp displacement at peak load, and energy per crack surface (fracture energy) in porcine and TAA human aortas.

### Clamp force-normalized displacement curves and fracture energy

**Fig. 1:**
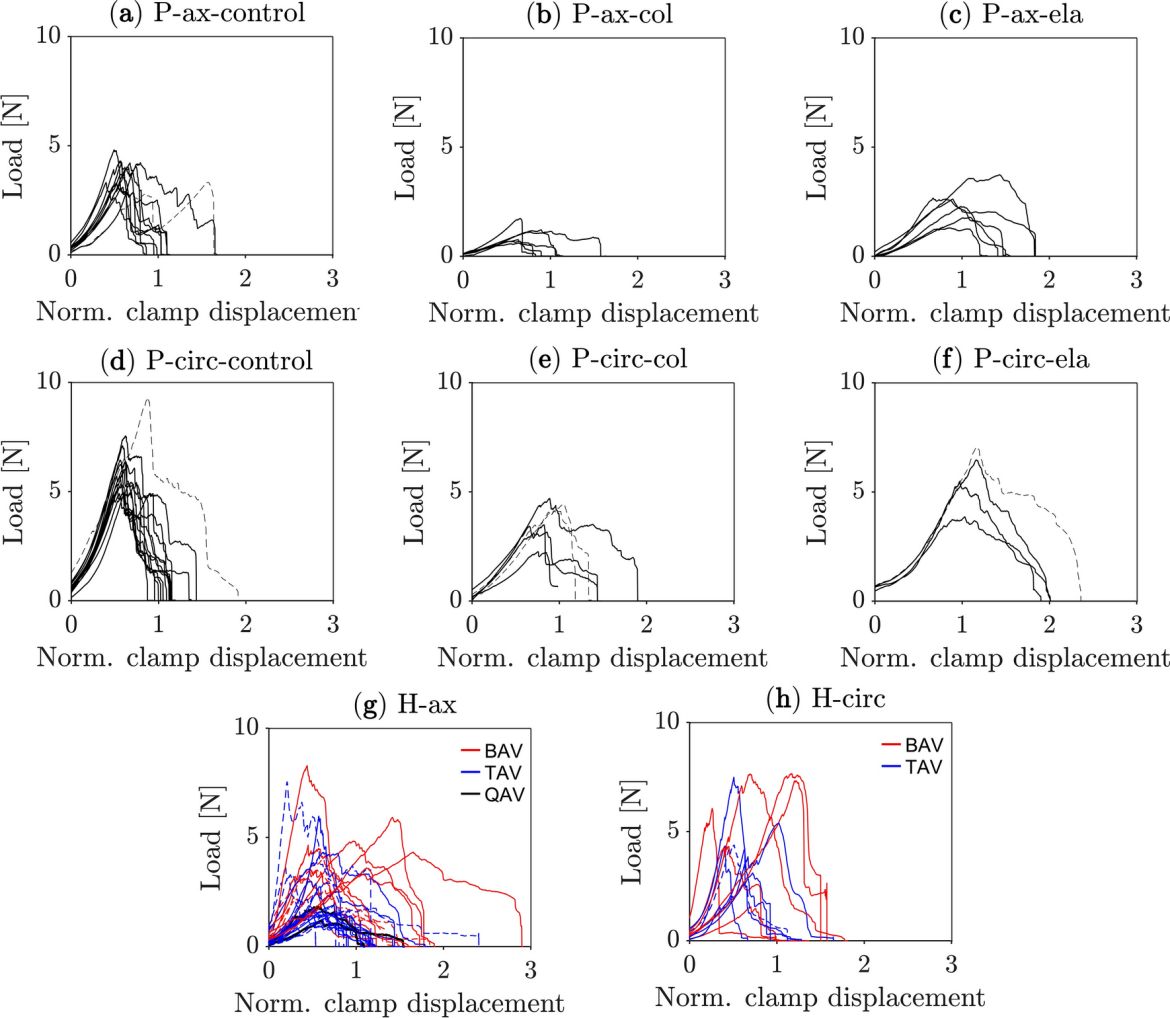
Clamp load vs. normalized displacement of the aorta recorded by the symmetry-constraint Compact Tension (symconCT) test. (**a**-**f**) Porcine tissue under axial (P-ax) and circumferential (P-circ) loading. Dashed curves refer to outliers. (control: normal aortic tissue, col: collagenase-treated tissue, ela: elastase-treated tissue). (**g**, **h**) Human tissue under axial (H-ax) and circumferential (H-circ) loading of the aneurysmatic thoracic aorta; red: aortas with bicuspid aortic valve (BAV); blue: aortas with a tricuspid aortic valve (TAV); black: aortas with a quadricuspid aortic valve (QAV). Dashed curves refer to tests that failed before the specimen has been completely separated.

**Fig. 2:**
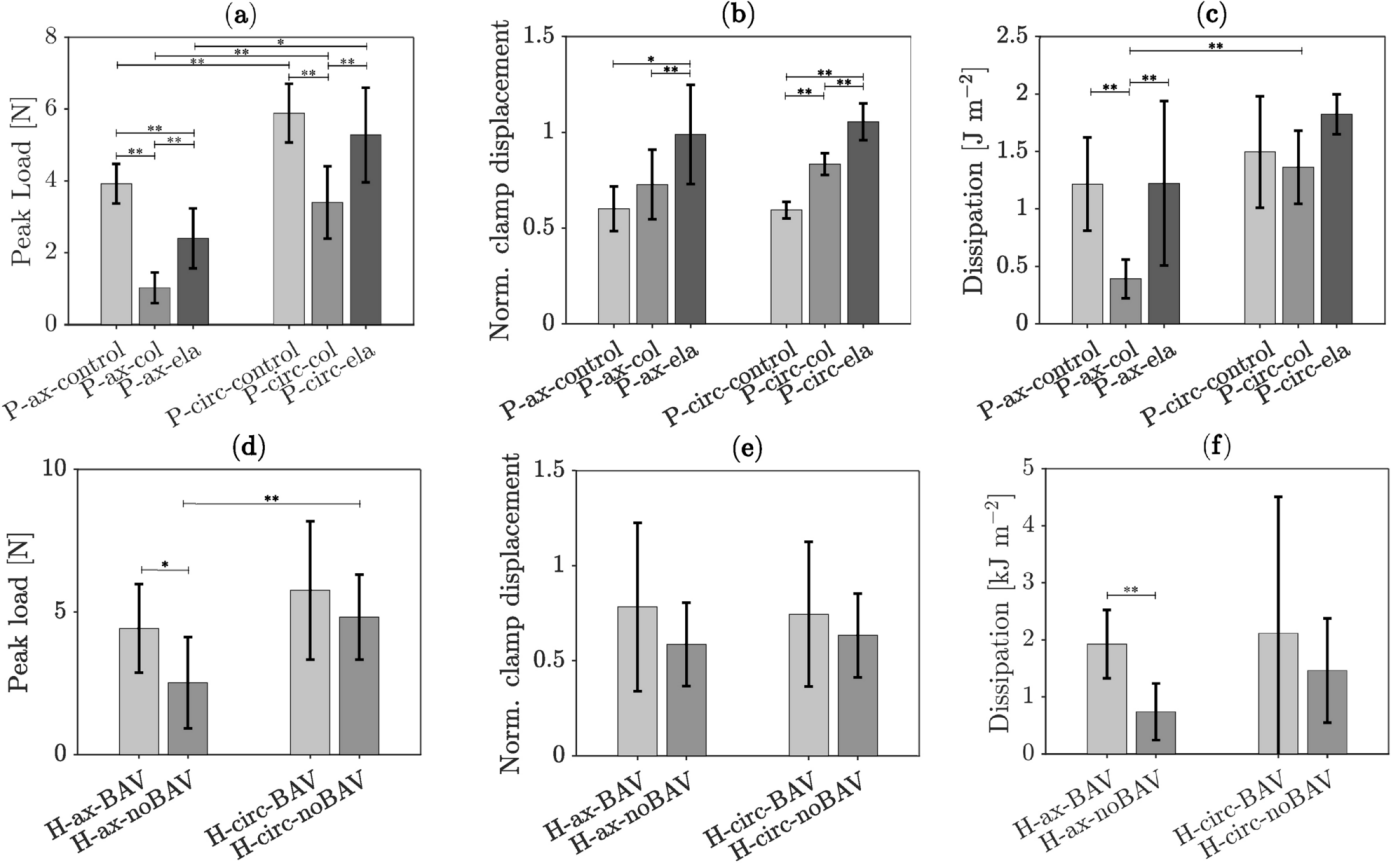
Mean and standard deviation of selective mechanical parameters characterizing porcine (**a**-**c**) and human (**d**-**e**) aortic tissue as recorded by the symmetry-constraint Compact Tension (symconCT) test. (**a**, **d**) Peak load. (**b**, **e**) Normalized clamp displacement at peak load. (**c**, **f**) Total fracture energy per unit undeformed fracture surface. (P: porcine; H: human; ax: axial loading; circ: circumferential loading; control: normal aortic tissue; col: collagenase-treated tissue, ela: elastase-treated tissue; BAV: Aorta with bicuspic aortic valve; noBAV: Aortas with valve other than BAV. * p-value < 0.05; ** p-value < 0.005).

Figure 1 illustrates the clamp force as a function of the normalized clamp displacement (strain) of porcine and human aortas loaded in axial and circumferential directions, respectively. As the specimen is pre-stretched, the force starts at a pre-load level and then increases until the peak load is reached. The force thereafter decays until the specimen is completely separated. Note that the crack may have already started to propagate prior to the peak load has been reached. Overall, the load variability recorded from porcine specimens was much lower than in human specimens. Loading along the circumferential direction led to higher forces in porcine and human specimens (p-value < 0.05), except for BAV cases; see Supplementary Table S1 and Fig. 2. Dashed curves in Fig. 1 refer to either outliers (porcine tissue) or tests that fell through after the peak load (human tissue).

Enzymatic digestion of ECM proteins in the porcine tissues affected the mechanical properties of the aortic wall. Particularly under axial loading, elastase and collagenase treatment caused a decrease in peak force by 39% and respectively 74% (elastase p-value = 1.25 10^–4^, collagenase p-value = 2.19 10^–8^, Fig. 2(a-c)). Given circumferential loading, only the collagenase-treated specimens exhibited a reduction of 42% in peak load (p-value = 2.44 10^–5^). In addition, enzymatically treated specimens needed more deformation to reach the peak load under both axial and circumferential loading (p-value = 4.82 10^–4^ and 3.16 10^–12^, respectively). Collagenase treatment reduced the energy per crack surface $D$ by 68% under axial loading (control:1.22 $\pm$ 0.41 kJ m^−2^; collagenase-treated: 0.39 $\pm 0.17$ kJ m^−2^; p-value = 2.47 10^–5^). Such a reduction was not observed under circumferential loading. Lastly, the loading direction influenced $D$ only for collagenase-treated porcine specimens (p-value = 2.29 10^–3^), and more results are listed in Supplementary Table S1 to Supplementary Table S5.

Regarding human aortas, 40% of tests fell through before the test specimen was completely separated, see Fig. 1(g, h) and Supplementary Figure S1. The specimen either slipped from the clamps or the rupture did not start at the pre-notch, both rendering a test unsuccessful. Such events were less frequent in porcine tissues (< 20%). As the computation of the fracture energy $D$ required completely separated test specimens, our data stems from, in total, only 25 tests (6 H-ax-BAV, 3 H-circ-BAV, 11 H-ax-noBAV, 5 H-circ-noBAV). Under axial loading, the BAV cases reached higher peak loads (4.42 $\pm$ 1.56 N, p-value = 5.15 10^–3^) and required more energy (1.92 $\pm$ 0.60 kJ m^−2^, p-value = 2.63 10^–3^) to rupture the specimen as compared to tissue from the noBAV cases (2.51 $\pm 1.60$ N and 0.74 $\pm 0.50$ kJ m^−2^). In addition, the noBAV cohort reached a higher peak load (4.82 $\pm$ 1.49 N, p-value = 2.66 10^–3^) and fracture energy (1.46 $\pm$ 0.91 kJ m^−2^, p-value = 0.052) when loaded in the circumferential as compared to the axial direction, respectively. See also Supplementary Table S1 and Supplementary Table S6.

For completion, the force–displacement curves of tests on human tissue, which were unsuccessful and hence disregarded in the data analysis, are reported in Supplementary Figure S1.

### The mechanical properties depend, at least partly, on the collagen content

**Fig. 3:**
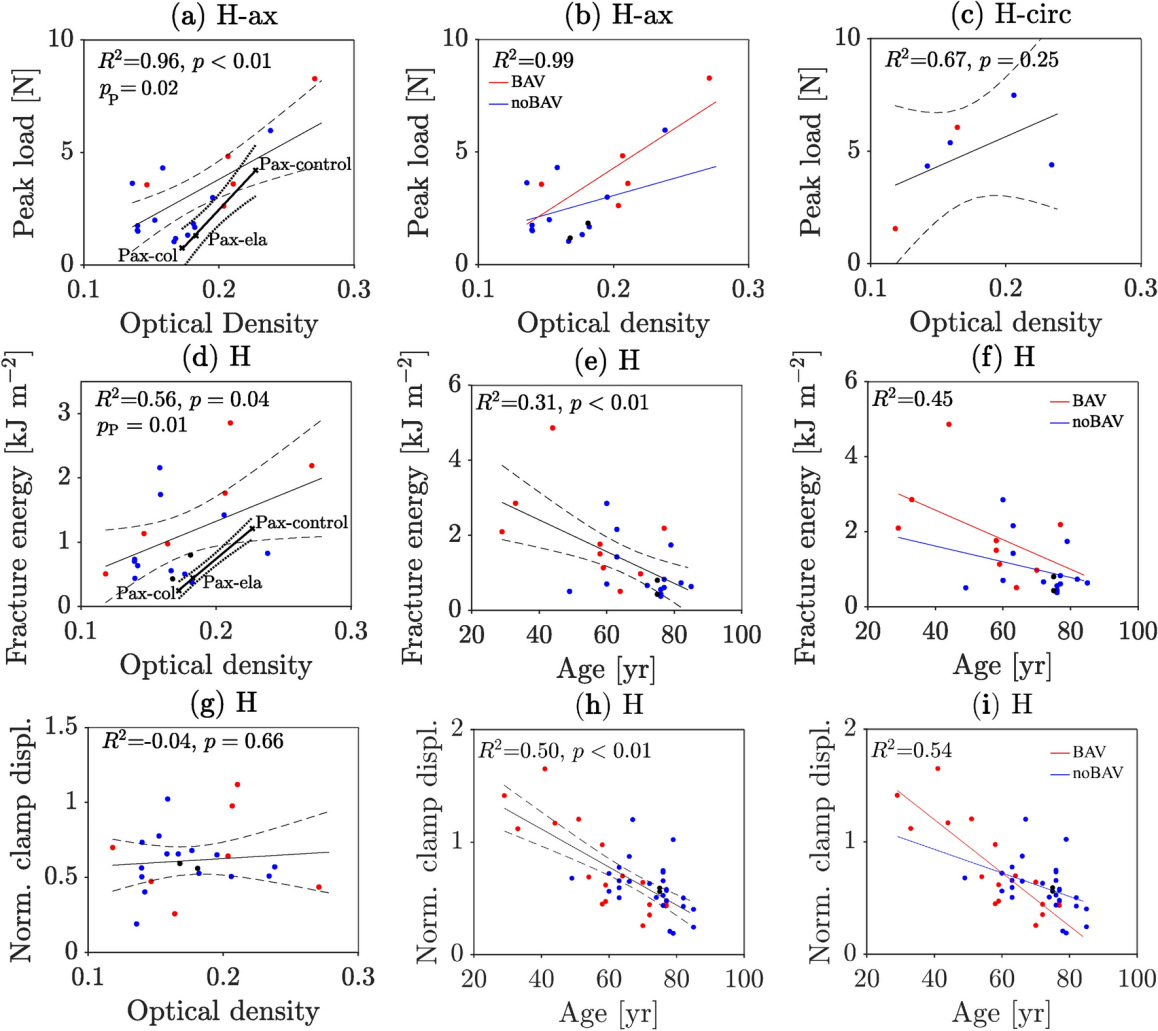
Influence of collagen optical density (OD) and patient age on aortic wall mechanical parameters as recorded by the symmetry-constraint Compact Tension (symconCT) test. (**a**, **b**, **c**) Peak load. (**d**, **e**, **f**) Fracture energy. (**g**, **h**, **i**) Normalized clamp displacement at peak load. Blue dot: Data from TAV patients. Red dot: Data from BAV patients. Black dot: Data from QAV patient. Black lines: analysis of BAV and noBAV cases when merged. The analysis is based on linear mixed-effects modelling (LME) with confidence bounds indicated by dashed lines. The ${R}^{2}$ is adjusted by the number of fixed effects. (P: porcine; H: human; ax: axial loading; circ: circumferential loading; control: normal aortic tissue; col: collagenase-treated tissue, ela: elastase-treated tissue; BSA: body surface area). Given human data, the p-value refers to the influence of the variable in the x-axis on the y-axis.

For axially loaded human aortas, the peak load ${P}_{\text{peak}}$ correlated positively with the collagen staining intensity in terms of average optical density (OD) (Fig. 3(a), ${R}^{2}$ = 0.96, p-value = 1.31 10^–3^ H-ax group). Collagen content also contributed to the variation of the peak load. In H-ax-noBAV cases and H-ax-BAV cases the normalization of ${P}_{\text{peak}}$ by OD reduced the standard deviations from approximately 62% to 54% and from 48 to 32%, respectively. Figure 3(b) shows the peak load ${P}_{\text{peak}}$ in relation to the average OD and the aortic valve anatomy (BAV, noBAV). Specifically, the statistical model considers the interaction by the respective equation in Supplementary Table S7. Although the average OD was unrelated to ${P}_{\text{peak}}$ for circumferentially loaded specimens, see Fig. 3(c), a similar trend to Fig. 3(a) can be noticed. Albeit not significant, this was also observed in the porcine aortic wall.

The fracture energy *D* of human tissue correlated positively with OD (Fig. 3(d), ${R}^{2}$=0.56, p-value = 0.04). However, only in the BAV cases under axial loading, normalizing *D* by OD reduced its standard deviation (from 37 to 29%; see Fig. 3(d)).

Lastly, the normalized clamp displacement at peak load was independent of OD; see Fig. 3(g). As the loading direction did not affect the normalized clamp displacement and *D*, circumferentially and axially loaded specimens have been considered together here.

### Mechanical properties are strongly associated with patient age

The patient’s age correlated negatively with the fracture energy *D*, see Fig. 3(e, f). Especially in BAV cases, $D$ appears to decrease quickly with age. By normalizing $D$ by the inverse of age, the standard deviation reduced from 65 to 57% in BAV cases and from 74 to 66% in noBAV cases, respectively. Specimens from older patients were, in general, stiffer, and reached ${P}_{\text{peak}}$ at lower applied clamp displacement, see Fig. 3(h, i). In addition, the standard deviation of the normalized clamp displacement reduced from 53 to 34% when normalized by the inverse of age of the BAV patients. Nevertheless, ${P}_{\text{peak}}$ was unaffected by the patient’s age (p-value = 0.42 in Hax group).

**Fig. 4:**
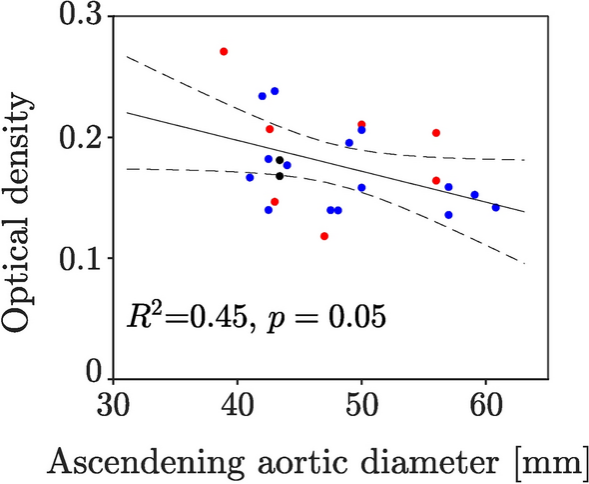
Change of collagen staining intensity in terms of optical density (OD) with the maximum diameter of the ascending aorta. Blue dot: Data from the thoracic aorta of TAV patients. Red dot: Data from the thoracic aorta of BAV patients. Black dot: Data from QAV patients. BAV and noBAV data are here grouped together. The analysis is based on linear mixed-effects modelling (LME) with confidence bounds indicated by dashed lines. The ${R}^{2}$ is adjusted by the number of fixed effects. Number of data = 24.

While the average OD did not correlate with the patient’s age, see Supplementary Figure S2, it decreased with increasing maximum diameters of the ascending aorta (${R}^{2}$ = 0.45, p-value = 0.05), see Fig. 4.

Clinical variables in relation to the measured mechanical properties are reported in Supplementary Table S8 to Supplementary Table S11. NoBAV specimens from patients with a history of myocardial infarction (MI) reached a higher peak load, a statement that was statistically significant only under axial loading (p-value = 5.02 10^–5^); see Supplementary Table S9. However, this trend was not observed in the BAV cases; see Supplementary Table S8. On the contrary, BAV cases with hypertension (HT) and BAV smokers resulted in a stiffer response, given a lower normalized clamp displacement at peak load (HT: p-value = 0.016, smoker: p-value = 0.006). Also, a lower normalized clamp displacement at peak load appeared associated with aortic insufficiency (AI) in BAV cases (p-value = 0.015).

### Fracture follows the circumferential direction more closely in non-normal tissue

**Fig. 5:**
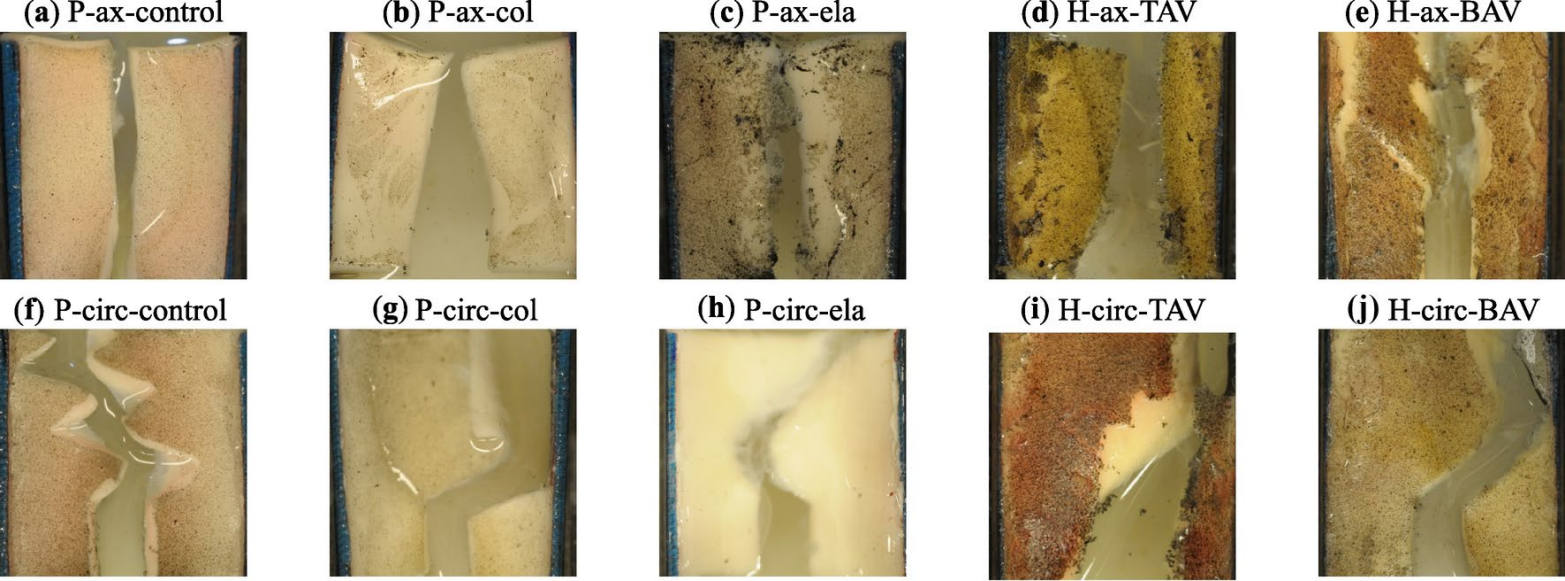
Crack patterns in porcine and human aortic tissue subjected to symmetry-constraint Compact Tension (symconCT) tests under axial (first row) and circumferential loading (second row). (P: porcine; H: human; ax: axial loading; circ: circumferential loading; control: normal aortic tissue; col: collagenase-treated tissue, ela: elastase-treated tissue; BAV: aorta with bicuspid aortic valve; TAV: aortas with tricuspid aortic valve).

**Fig. 6:**
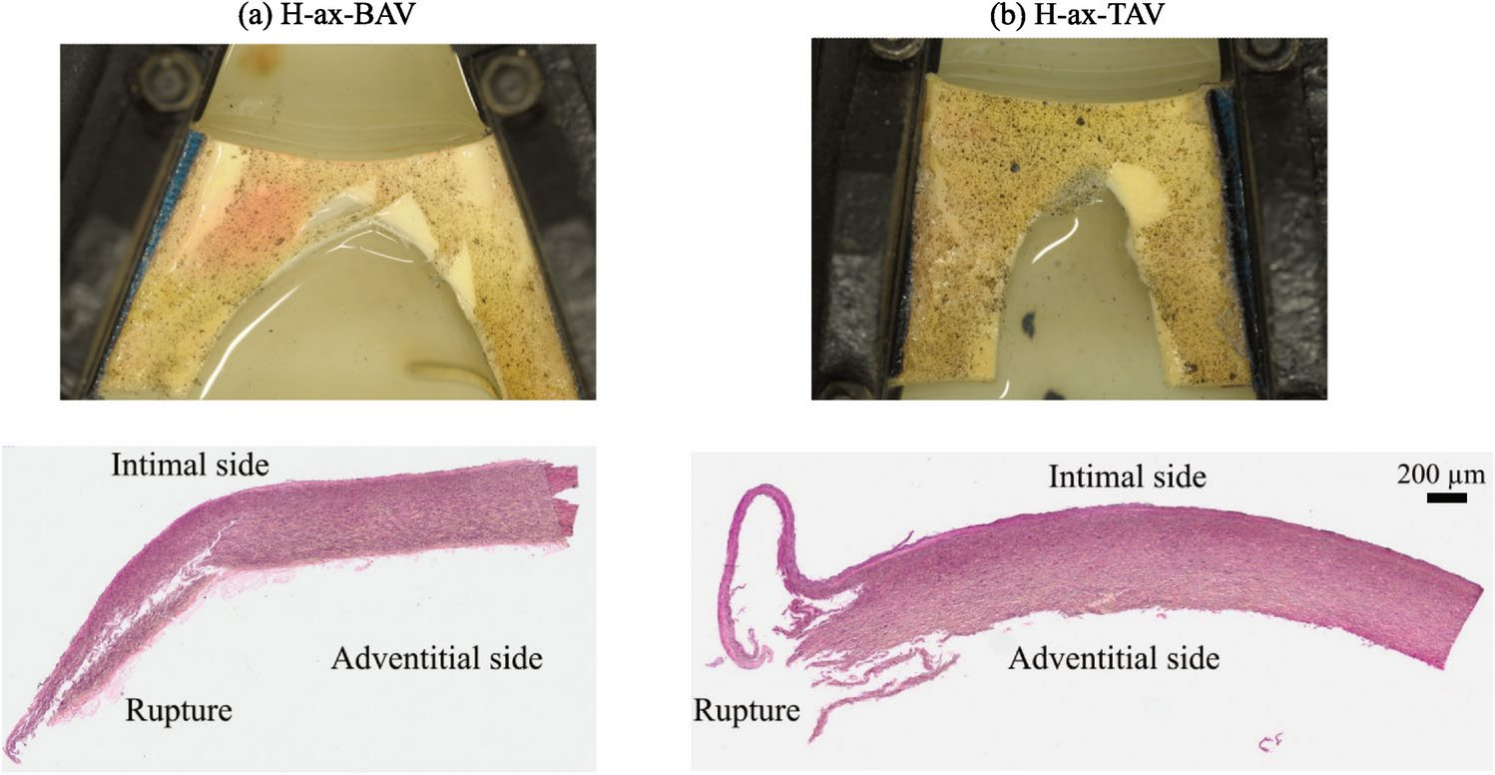
Fracture propagation during the symconCT experiment under axial loading of the TAA human specimens and corresponding Elastica van Gieson staining of representative cross-sections after testing; (**a**) TAA patient with bicuspid aortic valve (H-ax-BAV); (**b**) TAA patient with tricuspid aortic valve (H-ax-TAV).

Figure 5 illustrates the crack patterns of porcine and human aortic tissue specimens. In control as well as enzyme-treated porcine specimens, cracks consistently appeared straight under axial loading, as shown in Fig. 5. Here, the load was perpendicular to the circumferential direction, and thus the direction along which most collagen fibers are oriented. Likewise, human specimens under axial loading tended to rupture along a straight path. More interestingly, porcine controls under circumferential loading exhibited a repeated zig-zag crack pattern. In contrast, elastase-treated porcine specimens showed more disruptive tissue with less distinct crack deflections. Collagenase-treated porcine specimens had cracks that propagated toward the clamp, often at an angle of approximately 45°, and in two cases, the crack deflected in the opposite direction before reaching the clamp. Similar to the collagenase-treated specimens, most human tissue specimens displayed cracks that tilted towards the clamp under circumferential loading. Histological images revealed that the crack extended deep into the tissue, see Fig. 6. The vessel wall appeared less intact and compromised, with the intima and individual layers of the media rupturing in different ways. This led to the shear-related dissection and delamination of aortic layers during the symconCT test.

## Discussion

This work investigated the mechanical fracture properties of diseased human aortic tissue through the recently developed fracture symconCT experiment. Human specimens were analyzed with respect to aortic valve cuspidity, presenting well-known differences in molecular mechanisms of aneurysm development^10^. Comparisons with porcine aortic tissue tests and associations with clinical variables were analyzed in relation to high variability in fracture energy, peak load, and clamp displacement across 31 human aortic specimens. With variable amounts of energy for crack-formation and propagation, the present results align with the reported heterogeneity of TAA, *i.e.*TAV- or BAV-associated^39,40^. Our findings specifically indicate that the patient’s age, as well as the content of collagen fibers, influence the mechanical properties of the vessel wall, with these correlations being more pronounced in patients with a BAV. Our data demonstrate that with age, the aortas reached the peak load at lower levels of deformation and a low amount of energy was required to rupture the tissue. Ageing is accompanied by histopathological changes with regional differences, contributing to aorta stiffening^41,42^. Typically, ageing results in a relative loss of elastin integrity and content in human arteries while other matrix materials, primarily collagen, increase^41,43^. The loss of elastin integrity may cause localized structural changes, potentially leading to defects or microcracks that affect the fracture properties of aortic tissue.

In our cohort, the collagen OD value was not associated with aortic valve cuspidity but was instead correlated with the maximum diameter of the ascending aorta. Yet, the mechanical properties of BAV individuals appeared more sensitive to collagen OD. Notably, most clamp force-normalized displacement curves for TAV and QAV patients were similar, with only tests from two TAV aortas exceeding 2.5 N. In contrast, the BAV tests clearly differed across patients. This variability in BAV results may be influenced by the broader range of clinical characteristics, such as age and comorbidities, among BAV patients compared to those with TAV or QAV.

In general, tests on TAA human tissues produced mechanical responses similar to enzymatic-treated porcine specimens and, thus, to tissue with partially damaged collagen. Therefore, the quality of the collagen, and possibly of the collagen cross-linking^11^, might have been impaired in the TAA wall.

In particular, the peak load and the fracture energy needed to rupture are strongly linked to the collagen fibers, assessed in terms of OD. Aortic tissue with a lower OD, *i.e.* lower collagen content, easily ruptures; the tissue fails at lower forces, and a crack surface can be formed at less fracture energy. Other histological constituents, such as cells and glycosaminoglycans, may also influence fracture propagation. However, as collagen plays a major load-bearing role at stress levels relevant to fracture, it might be considered the most crucial factor in determining when and how a fracture propagates.

Unlike most porcine specimens, visible dissection and tearing of the human aortic intima and media layers occurred during the experiment; see Fig. 6. The fracture pattern in human tissue was, therefore, more diffuse, and beside the primary crack, secondary fractures—likely caused by shear-dominated failure mechanisms—determined the vessel wall rupture. Macroscopic observations are supported by histological analysis, which clearly showed irreversible deformation (damage) deep inside the tissue. Similar delamination/dissection events have only been seen in a few treated porcine specimens, especially the elastase-treated ones. As the loss and fragmentation of elastin fibers are characteristic features of TAV-associated TAA, but not BAV-TAA^42^, it may have contributed to the observed disruption and delamination of elastic lamellae in the media. In addition, the decrease in collagen, as measured by OD could have reinforced the formation of secondary fracture features.

Dissection energy has been previously quantified and varied from 0.0165 kJ m^−2 ^for human aortas^46^ to 0.159 kJ m^−2 ^for upper descending porcine aortas^47^. Dissection of human aortas induced by a peeling test^36^ resulted in an energy of 0.076 kJ m^−2^, and a more recent investigation identified very low energy levels of 0.005 kJ m^−2^ (mode II) and 6.31 10^–4^ kJ m^−2 ^(mode I), for the dissection-like failure of ascending sheep aortas^27^. As dissection likely eases fracture propagation, it explains that the fracture energy identified in the present study was orders of magnitude higher. Intrinsic toughening mechanics ahead of the crack tip, such as crack tip blunting, fiber sliding and fiber debonding can increase the material’s toughness and affect the crack path. Any changes to the material constituents and structure can impact these intrinsic toughening mechanisms, mitigating crack deflections. We, therefore, assume that the orientation, quality, and quantity of collagen fibers play a crucial role in the formation of the crack path and especially its deflections. A tortuous crack path developing during the symconCT test potentially indicates a healthier vessel wall. It is supported by the observation that the collagenase-treated porcine specimens as well as all human specimens, seldom exhibited crack path deflections under circumferential loading.

In our study, the discussion of fiber orientation is based on the common understanding of vascular collagen organization given literature observations^14^.

The strength of our conclusions is limited by the small amount of data, particularly in the human tissue tests, which were often unsuccessful. Additional data may help to clarify clinical factors that influence the mechanical properties of the aorta. Furthermore, the analysis was complicated by the significant variability in results due to the lack of pre-conditioning of the test specimens and the vastly different clinical conditions. A key limitation of the symconCT test is its inability to replicate biaxial in-vivo loading, as well as the inability to monitor tissue damage before fracture propagation. The specimen size and loading rate may also influence the fracture properties, suggesting that future research should investigate these effects. Additionally, the fracture mechanical properties in vivo might differ from those in our study, as the tissue samples were frozen prior to testing. As the elastic tissue properties seem not to be affected by freezing^48^, we might expect the same for the fracture properties, an assumption, however, worth validating in future studies. Another challenge was that the human and porcine tissue specimens differed in size, as some human aortas were smaller. Despite this, a cross-species comparison of the ascending aorta remains difficult and, to our knowledge, has not yet been systematically explored in the context of rupture formation and propagation.

## Methods

### Specimen preparation and test set-up

#### Porcine cohort

Porcine aortas (n = 11) were purchased from a local slaughterhouse and transported on ice to the laboratory. The aortas were frozen at −20°C. Prior to testing, the aortas were thawed at 4°C in saline solution for approximately 24 h and processed at room temperature. During its preparation, the specimen was periodically moistened with saline solution. Any loose connective tissue was removed, and each aorta was cut open longitudinally. As our objective was the testing of the media-intima compound, the adventitia layer was carefully dissected away under a magnification loupe.

Besides exploring the native tissue, collagenase- and elastase-treated test specimens were prepared. The supplied collagenase powder (245 U mg^−1^, Type II, Worthington Biochemical Corporation) and the lyophilized elastase (4.24 U mg^−1^, Worthington Biochemical Corporation) were diluted in physiological solution at concentrations of 500 U ml^−1^ and 5 U ml^−1^, respectively. Aortic specimens were then incubated at 37°C for 6 h (BioTester 5000. CellScale, Inc.) and washed thoroughly with saline solution (distilled water with 0.9% NaCl) before mechanical testing.

#### Human cohort

Ascending aortic specimens from 36 patients were collected as part of the DAVAACA study^13,49^; see Table 1. All patients (7 women, 29 men; age: 63.7 ± 14.9(SD) yr, range 27–85 yr) had undergone elective open‐heart surgery for ascending aortic aneurysm at the Karolinska University Hospital, Solna, Sweden. The study was approved by the Regional Research Ethics Approval Committee in Stockholm (application no. 2012/1633‐31/4) and was conducted in accordance with the Declaration of Helsinki. Written consent was obtained from all individuals. The maximum ascending aortic diameter was measured intraoperatively by transesophageal echocardiography, from leading edge to leading edge in end‐diastole at the level of the ascending aorta. Body surface area was calculated using the Mosteller formula, and height and weight measurements were recorded preoperatively. Medications and comorbidities were self‐reported via a study questionnaire, which was completed with a research nurse.

**Table 1:** Clinical parameters of the patients associated with the human aortic wall samples.

Clinical parameter		H-noBAV n = 20	H-BAV n = 16
Female	Female sex rate	25%	12.50%
MI	Myocardial Infarction	25%	12.50%
CADS	Coronary artery disease, bypass surgery, percutaneous intervention	30%	18.75%
Smoker	Cigarette or pipe smoker in the past or present time	65%	43.75%
Diabetes		10%	6.25%
HT	Hypertension	75%	31.25%
HF	Heart failure or transient ischemic attack	30%	6.25%
AI	Aortic insufficiency	60%	43.75%
AS	Aortic stenosis	0	37.50%
Ectasia		35%	25%
AAA	Abdominal Aortic Aneurysm	30%	18.75%
BAV	Bicuspid aortic valve	0	100%
TAV	Tricuspid aortic valve	95%	0
QAV	Quadricuspid aortic valve	5%	0
BSA [m^2^]	Body surface area	1.93(0.26)	2.02(0.20)
Ascendens [mm]	Ascending aortic diameter	46.81(6.95)	45.02(5.66)
Age [yr]	Age of the patient at surgery	69.6(13.2)	56.3(13.8)

H-noBAV: human patients having either a tricuspid or a quadricuspid aortic valve; H-BAV: patients having a bicuspid aortic valve. Mean and standard deviation (SD) are reported for BSA, ascendens, and age.

Upon harvesting, the aortas were stored in sterile saline solution at −80°C until transportation to the biomechanical laboratory, where they were kept at −20°C and tested within a month. Following the above-described protocol of porcine tissue preparation, the human tissue was thawed, and the adventitia layer was dissected.

#### Specimen preparation

**Fig. 7:**
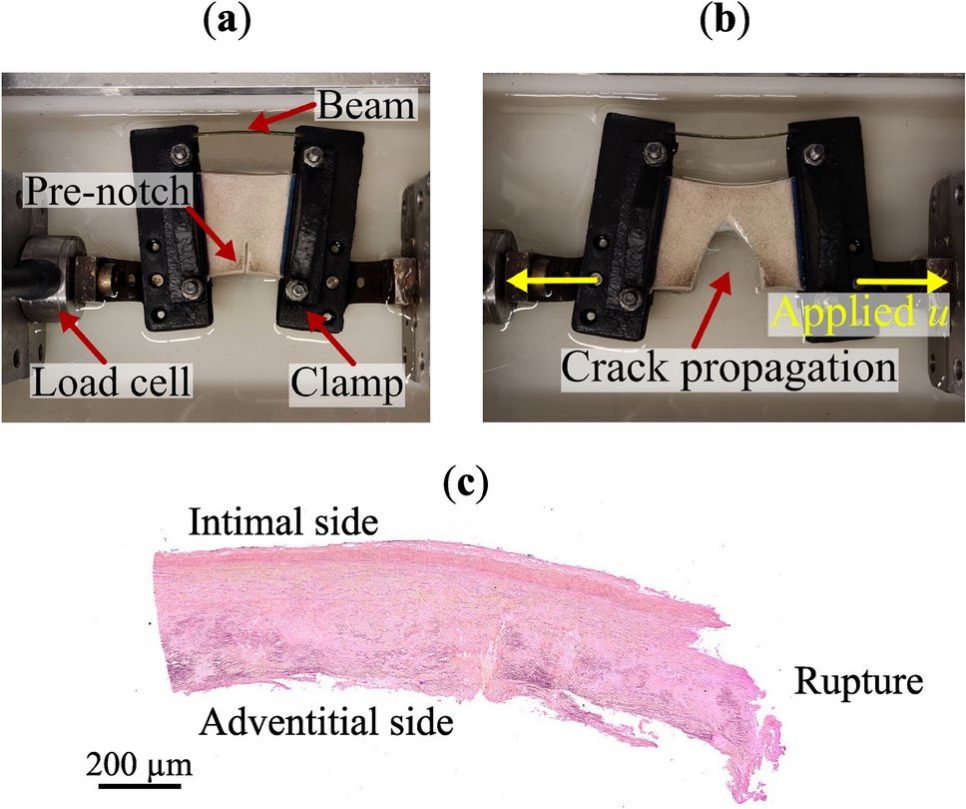
Porcine aorta specimen that is mechanically unloaded (**a**) and loaded (**b**) in the symmetry-constraint Compact Tension (symconCT) test. (**c**) Elastica Van Gieson staining on human tissue after symconCT testing.

On average, 35 × 30 mm^2^ and 28 × 30 mm^2^ large specimens for mechanical testing were prepared from porcine and human tissue, respectively. Along the 30 mm long side, sandpaper was glued (Loctite super glue) to the specimen to prevent slipping in the clamps during mechanical testing. Using a surgical scalpel, a cut (pre-notch) was introduced in the test specimen and perpendicular to the anticipated direction of loading; see Fig. 7. These cuts were, on average, 10 mm and 11 mm long in porcine and human specimens, respectively. Prior to testing, the precise specimen dimensions and the pre-notch length were measured with a caliper (Absolute Digimatic Caliper, Mitutoyo America Corporation) and by analyzing macroscopic images (ImageJ2, version 2.9.0). The average specimen thickness was acquired by sandwiching the specimen between glass plates. Particular care was taken to avoid any squeezing of the tissue. Finally, a homogeneous, randomly distributed black speckle pattern was sprayed onto the surface of the intima layer to allow for the DIC analysis. Specimens were labelled according to the origin of the aorta, either ‘P’ (porcine) or ‘H’ (human), and to the loading direction, either ‘ax’ (axial direction of the vessel’s wall) or ‘circ’ (circumferential direction of the vessel’s wall). The analysis was performed considering specimens from patients having BAV or TAV (H-ax, H-circ) and also excluding the BAV cases (H-ax-noBAV, H-circ-noBAV). The porcine specimens were also labelled as ‘ela’ (elastase-treated), ‘col’ (collagenase-treated) or ‘control’ (no enzymatic treatment).

#### SymconCT testing protocol

The Compact Tension (CT) test is the standard test protocol to study fracture properties in metallic and a variety of other materials (ASTM E647-00, ISO 7539–6:2003). A tensile force is applied to a notched specimen, causing a mode I-dominated opening fracture. However, the classical CT test is not suitable for use with soft tissue, as the specimen would buckle opposite to the notch, and the test would fail. Therefore, the classical Compact Tension test was augmented to enable stable fracture propagation, resulting in the symmetry-constraint Compact Tension (symconCT) test, a suitable set-up for fracture testing of vascular tissue. An elastic beam (high-strength carbon steel) connects the two clamps, promoting a symmetric opening of the clamps. It additionally prevents the specimen from buckling by applying a pre-stretch of approximately 1.13 and 1.26 in the porcine and human tissues, respectively.

The mechanical tissue characterization was performed in 0.9% sodium chloride solution at 37°C using a uniaxial tensile system (ADMET eXpert 4000 Universal Testing System) at a clamp displacement rate of 3 mm min^−1^ and respectively 2 mm min^−1^ for the porcine and human specimens, corresponding to a clamp strain rate of 0.002 s^−1^. The force and clamp displacement were recorded until the specimen was completely separated. The test was combined with Digital Image Correlation (DIC) (Vic-2D, Correlated Solutions, Inc.), where a digital camera (Nikon D300S equipped with a lens Sigma 105 mm F2.8 DG DN MACRO, Sigma Corporation) captured images from porcine and human specimens at 1.0 and 0.5 Hz, respectively.

### Biomechanical and histological parameters extraction

To provide a better comparison across porcine and human specimens, the recorded clamp load $F$ versus displacement $u$ data was translated into force versus normalized displacement (strain) $\varepsilon = u/A$ curves, where $A$ denotes the distance between the clamps of the unloaded specimen.

Given the recorded force–displacement curves, we computed the external work ${W}_{\text{ext}} =\int Pdu$ needed to perform the test, where $P$ and $u$ denote the reduced clamp force and the clamp displacement, respectively. Here, *P* represents the force recorded by the load cell and reduced by the contribution related to the bending of the elastic beam of the symconCT test set-up. For comparison purposes, the external work was normalised with the fracture surface, resulting in a referential fracture energy $D = {W}_{\text{ext}}/(BL)$, where $B$ and $L$ denote the specimen thickness and crack length in the undeformed configuration, respectively. In particular, the referential crack length was obtained using multiplicative kinematic decomposition as described elsewhere^50^. It mapped the crack path from the pre-stretch state, available from the DIC recordings, onto the referential undeformed configuration. Besides the work required to form the fracture surface $BL$, the energy $D$ also includes energy dissipated by other irreversible means.

#### Histological analysis

Tissue was obtained from the fractured symconCT-tested specimens, embedded in paraffin and sectioned at 5 µm using a microtome (Microm HM 355S, Thermo Scientific). Subsequent staining with Masson trichrome and Elastica Van Gieson was performed according to manufacturer protocols. Digital images of each section were attained with a digital slide scanner (VS200, Olympus). A trained physician evaluated Elastica Van Gieson’s stained aortic sections for degrees of medial degeneration in accordance with consensus on surgical pathology of the aorta^42^. To study the histological collagen estimation in each image, a deconvolution method was employed in ImageJ2 (version 2.9.0), isolating the methyl blue stain in Masson trichome stained sections. A systematic random sample of > 20 equal-sized sub-regions of the intima and media per image was analyzed, and the average optical density (OD) of methyl blue staining computed.

### Statistical analysis

Despite morphological and biological differences between aortas with quadricuspid^44,45^ (QAV) and TAV, the aorta with QAV in our cohort exhibited mechanical properties similar to TAV cases. It was, therefore, added to the aortas with TAV, forming the non-BAV (noBAV) cohort. In addition, as the experiments from five individuals (see Supplementary Data) were unsuccessful, these data were removed from the analysis.

All quantitative data are presented as mean and standard deviation (SD), and the statistical analysis was performed (MATLAB R2024a, The MathWorks, Inc.). To investigate the normal (or log-normal) distribution, an Anderson–Darling test was performed, and any outlier from the porcine tests was identified by Grubb’s test function (GraphPad, GraphPad Software). The significance levels were set to p-value < 0.05.

The linear mixed-effects (LME) model with the restricted maximum likelihood method was used to investigate the influence of the loading direction, clinical factors and the collagen staining intensity as measured by the optical density of staining (OD) on mechanical parameters, such as peak load, clamp stretch at peak load, and fracture energy. Given more than one measurement was acquired from a single tissue source (porcine or human), the LME model considered the tissue source as a random factor.

The LME test was also used to explore regional dependence (proximal, central, distal) as well as the influence of enzymatic treatment (controls, collagenase-treated and elastase-treated specimens) on the results of the porcine specimens. Here, the animal from which the specimen originated was regarded as a random effect.

## Supplementary Information

Supplementary Information 1.

Supplementary Information 2.

## Data Availability

Correspondence and requests for materials should be addressed to T.C.G.
