# Supplementary material for: Fracture properties of porcine versus human thoracic aortas from tricuspid/bicuspid aortic valve patients via symmetry-constraint Compact Tension testing
Source: Sci Rep. 2025 Jan 3;15:667. doi: 10.1038/s41598-024-83233-6 (PMC11699116; doi:10.1038/s41598-024-83233-6)
Supplement: Supplementary file 1 — Supplementary Information 1. [file 41598_2024_83233_MOESM1_ESM.docx]

# Supplementary Material


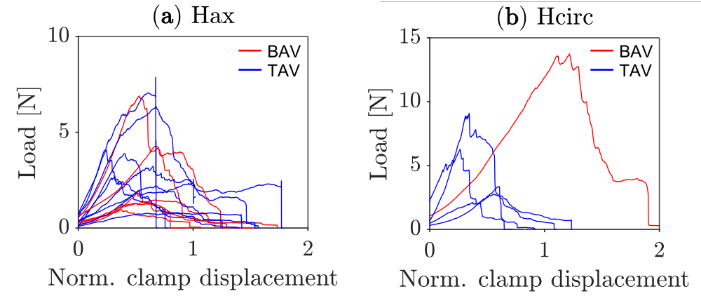


Supplementary Figure S1-Clamp load vs normalized displacement recorded by the symmetry-constraint Compact Tension (symconCT) tests, which failed due to either the sliding of the specimen or a rupture not at the pre-notch. The specimens are from aneurysmatic human thoracic aorta under axial (H-ax) and circumferential (H-circ) loading; red: aortas with bicuspid aortic valve (BAV); blue: aortas with a tricuspid aortic valve (TAV).

Supplementary Table S1-Influence of loading direction, axial (ax) versus circumferential (circ), on the porcine (P) and human (H-BAV: human with bicuspid aortic valve, H-noBAV: human with tricuspid or quadricuspid valve) tissue properties from the symconCT fracture experiments. Except for the controls (-control), the porcine specimens underwent elastase (-ela) or collagenase (-col) treatment. The table shows the number of data (n) and p-values of the Linear Mixed Effect ( LME) model analysis. $P_{\mathrm{peak}}$, $\varepsilon_{P_{peak}}$, $D$, and$OD$ denote the peak load, the normalized clamp displacement at the peak load, the fracture energy, and the optical density of staining, respectively.

| **Variable**  ax vs circ | **Influence of loading direction**  **p-value (LME)** | | | | |
| --- | --- | --- | --- | --- | --- |
|  | **P -control** | **P -col** | **P -ela** | **H-BAV** | **H-noBAV** |
| $P_{\mathrm{peak}}$ | $\boldsymbol{2.785 1}\boldsymbol{0}^{\boldsymbol{-6}}$  n=24 | $\boldsymbol{4.912}\boldsymbol{10}^{\boldsymbol{-3}}$  n=10 | $\boldsymbol{0.012}$  n=9 | $0.184$  n=17 | $\boldsymbol{2.661 1}\boldsymbol{0}^{\boldsymbol{-}\boldsymbol{3}^{\boldsymbol{*}}}$  n=29 |
| $\varepsilon_{P_{\mathrm{peak}}}$ | $0.860$  n=24 | $0.298$  n=10 | $0.683$  n=9 | ${0.603}^{*}$  n=17 | ${0.641}^{*}$  n=29 |
| $D$ | ${0.099}^{*}$  n=24 | $\boldsymbol{2.291 1}\boldsymbol{0}^{\boldsymbol{-3}}$  n=9 | $0.382$  n=24 | 0.905  n=9 | ${0.052}^{*}$  n=16 |
| OD |  |  |  | 0.095  n=7 | 0.131  n=17 |
| *^*^Log-normal distribution.* | | | | | |

Supplementary Table S2- Influence of the enzymatic treatments, elastase (-ela) versus collagenase (-col) versus controls (-control), on the porcine (P) aortic tissue properties from the symconCT fracture experiments, under axial (ax) and circumferential (circ) loading. The table denotes the number of data (n) and the p-values of the Linear Mixed Effect (LME) model analysis. $P_{\mathrm{peak}}$, $\varepsilon_{P_{\mathrm{peak}}}$ , and D denote the peak load, the normalized clamp displacement at the peak load, and the fracture energy, respectively.

| **Variable**  control vs col vs ela | **Influence of enzymatic treatment  p-value (LME)** | |
| --- | --- | --- |
|  | **P-ax** | **P-circ** |
| $P_{\mathrm{peak}}$ | $\boldsymbol{4.814}\boldsymbol{10}^{\boldsymbol{-7}}$  n=22 | $\boldsymbol{2.043}\boldsymbol{10}^{\boldsymbol{-4}}$  n=21 |
| $\varepsilon_{P_{\mathrm{peak}}}$ | $\boldsymbol{1. 938 1}{\boldsymbol{0}^{\boldsymbol{-3}}}^{\boldsymbol{*}}$  n=22 | $\boldsymbol{1.938}\boldsymbol{10}^{\boldsymbol{-11}}$  n=21 |
| $D$ | $\boldsymbol{9.336}{\boldsymbol{10}^{\boldsymbol{-5}}}^{\boldsymbol{*}}$  n=22 | $0.416$  n=20 |
| *^*^Log-normal distribution.* | | |

Supplementary Table S3- Pair-wise comparison of the influence of the enzymatic treatment treatments, elastase (ela) versus collagenase (col) versus controls (control), on the normalized clamp displacement at peak load measured from the symconCT fracture experiments on porcine (P) aortic tissue, under axial (ax) and circumferential (circ) loading. The table denotes the number of data (n) and the p-values of the Linear Mixed Effect (LME) model analysis.

| **Norm. clamp displ.** | **Influence of enzymatic treatment  p-value (LME)** | |
| --- | --- | --- |
|  | **P-ax** | **P-circ** |
| control vs col | ${0.127}^{*}$  n=6 | $\boldsymbol{9.778}\boldsymbol{10}^{\boldsymbol{-9}}$  n=18 |
| control vs ela | $\boldsymbol{4.817}{\boldsymbol{10}^{\boldsymbol{-4}}}^{\boldsymbol{*}}$  n=16 | $\boldsymbol{3.162}\boldsymbol{10}^{\boldsymbol{-12}}$  n=17 |
| ela vs col | $\boldsymbol{0.031}$  n=12 | $\boldsymbol{2.013}\boldsymbol{10}^{\boldsymbol{-5}}$  n=7 |
| *^*^Log-normal distribution.* | | |

Supplementary Table S4- Pair-wise comparison of the influence of the enzymatic treatment treatments, elastase (ela) versus collagenase (col) versus controls (control), on the peak load measured from the symconCT fracture experiments on porcine (P) aortic tissue, under axial (ax) and circumferential (circ) loading. The table denotes the number of data (n) and the p-values of the Linear Mixed Effect (LME) model analysis.

| **Peak load** | **Influence of enzymatic treatment  p-value (LME)** | |
| --- | --- | --- |
|  | **P-ax** | **P-circ** |
| control vs col | $\boldsymbol{2.191}\boldsymbol{10}^{\boldsymbol{-8}}$  n=6 | $\boldsymbol{2.442}\boldsymbol{10}^{\boldsymbol{-5}}$  n=18 |
| control vs ela | $\boldsymbol{1.246 1}\boldsymbol{0}^{\boldsymbol{-4}}$  n=16 | $0.069$  n=17 |
| ela vs col | $\boldsymbol{0.002}$  n=12 | $\boldsymbol{2.302}\boldsymbol{10}^{\boldsymbol{-3}}$  n=7 |

Supplementary Table S5- Pair-wise comparison of the influence of the enzymatic treatment treatments, elastase (ela) versus collagenase (col) versus controls (control), on the fracture energy measured from the symconCT fracture experiments on porcine (P) aortic tissue, under axial (ax) and circumferential (circ) loading. The table denotes the number of data (n) and the p-values of the Linear Mixed Effect (LME) model analysis.

| **Fracture energy** | **Influence of enzymatic treatment  p-value (LME)** | |
| --- | --- | --- |
|  | **P-ax** | **P-circ** |
| control vs col | $\boldsymbol{2.469}{\boldsymbol{10}^{\boldsymbol{-5}}}^{\boldsymbol{*}}$  n=16 | $0.642$  n=17 |
| control vs ela | ${0.850}^{*}$  n=16 | $0.258$  n=17 |
| ela vs col | $\boldsymbol{3.946 1}{\boldsymbol{0}^{\boldsymbol{-4}}}^{\boldsymbol{*}}$  n=12 | $0.217$  n=6 |

Supplementary Table S6- Influence of bicuspid aortic valve (BAV) compared to tricuspid (TAV) or quadricuspid (QAV) valve on human tissue properties from the symconCT fracture experiments. $P_{\mathrm{peak}}$, $\varepsilon_{P_{peak}}$, $D$, and$OD$ denote the peak load, the normalized clamp displacement at the peak load, the fracture energy, and the optical density of collagen staining intensity, respectively. The table denotes the p-values and number of data (n) of the Linear Mixed Effect (LME) model analysis.

| **Variable**  BAV vs TAV/QAV | **Influence of BAV  p-value (LME)** | |
| --- | --- | --- |
|  | **H-ax** | **H-circ** |
| $P_{\mathrm{peak}}$ | $\boldsymbol{5.15}{\boldsymbol{10}^{\boldsymbol{-3}}}^{\boldsymbol{*}}$  n=34 | $0.44$  n=12 |
| $\varepsilon_{P_{\mathrm{peak}}}$ | ${0.089}^{*}$  n=34 | $0.547$  n=12 |
| $D$ | $\boldsymbol{2.63 1}{\boldsymbol{0}^{\boldsymbol{-3}}}^{\boldsymbol{*}}$  n=17 | $0.59$  n=8 |
| OD | $\boldsymbol{0.048}$  n=18 | $0.274$  n=6 |
| *^*^Log-normal distribution.* | | |

Supplementary Table S7- Linear Mixed Effect (LME) models utilized in the corresponding charts in Figure 3. Since more tests belonged to a single human sample, the patient ID was used as a random effect. The interaction between the fixed effects is denoted as ‘:’. The table denotes the p-values and the number of data (n) in the analysis.

| **Figure** | **LME model** | **Tests** | **p-value (LME)** |
| --- | --- | --- | --- |
| Figure 3(a) | $P_{\mathrm{peak}} \sim1+OD+(1\vert\mathrm{patient})$ | H-ax | $\boldsymbol{1.31}\boldsymbol{10}^{\boldsymbol{-3}}$  n=34 |
| Figure 3(b) | $P_{\mathrm{peak}} \sim1+OD+BAV+BAV:OD+(1\vert\mathrm{patient})$ | H-ax | OD:**0.032**, BAV:0.428, OD:BAV:0.273 n=34 |
| Figure 3(c) | $P_{\mathrm{peak}} \sim1+OD+(1\vert\mathrm{patient})$ | H-circ | 0.248  n=12 |
| Figure 3(d) | $D \sim1+OD+(1\vert\mathrm{patient})$ | H-ax and H-circ | **0.041**  n=19 |
| Figure 3(e) | $D \sim1+\mathrm{Age}+(1\vert\mathrm{patient})$ | H-ax and H-circ | $\boldsymbol{2.09 10}^{\boldsymbol{-3}}$  n=25 |
| Figure 3(f) | $D \sim1+Age+BAV+Age:BAV+(1\vert\mathrm{patient})$ | H-ax and H-circ | Age:0.058, BAV: 0.434, Age:BAV:0.569  n=25 |
| Figure 3(g) | ${\lambda_{P}}_{\mathrm{peak}} \sim1+OD+(1\vert\mathrm{patient})$ | H-ax and H-circ | 0.657 n=24 |
| Figure 3(g) | ${\lambda_{P}}_{\mathrm{peak}} \sim1+\mathrm{Age}+(1\vert\mathrm{patient})$ | H-ax and H-circ | $\boldsymbol{2.78 1}\boldsymbol{0}^{\boldsymbol{-8}}$  n=46 |
| Figure 3(g) | ${\lambda_{P}}_{\mathrm{peak}} \sim1+Age+BAV+Age:BAV+(1\vert\mathrm{patient})$ | H-ax and H-circ | Age:$\boldsymbol{4.72 1}\boldsymbol{0}^{\boldsymbol{-8}}$,  BAV: 0.052, Age:BAV:**0.028**  n=46 |


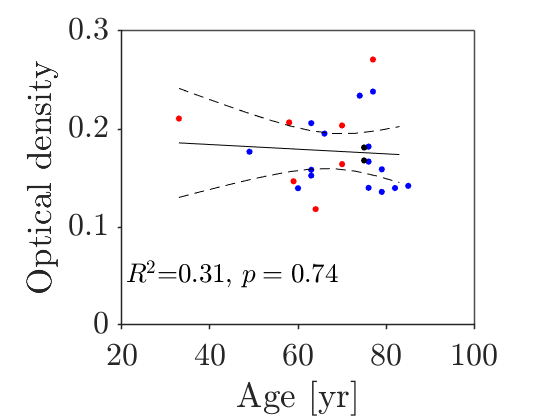


Supplementary Figure S2- Change of collagen staining intensity in terms of optical density (OD) with the patient’s age. Blue dot: data from the thoracic aorta of tricuspid aortic valve (TAV) patients. Red dot: data from the thoracic aorta of bicuspid aortic valve (BAV) patients. Black dot: data from a quadricuspid aortic valve (QAV) patient. BAV and noBAV data are here grouped together. The analysis is based on linear mixed-effects modelling (LME) with confidence bounds indicated by dashed lines. The R^2^ is adjusted by the number of fixed effects. Number of data = 24.

Supplementary Table S8- Mean, standard deviation (SD) of the peak load, and statistical influence of clinical parameters on the peak load on the human thoracic aortic specimens from patients having tricuspid or quadricuspid aortic valves (H-noBAV) under both axial and circumferential loading in the symconCT test. The table denotes the p-values of the Linear Mixed Effect (LME) model analysis, which were computed only for a number of data (n) >4. *Log-normal data distribution. The abbreviations of the clinical parameters are listed in Table 1.

| **Clinical Parameter** | **H-ax-noBAV "Yes"** | **H-ax-noBAV "No"** | ***p*-value (LME)** | **H-circ-noBAV "Yes"** | **H-circ-noBAV "No"** |
| --- | --- | --- | --- | --- | --- |
|  | $P_{\mathrm{peak}} [N]$ | |  | $P_{\mathrm{peak}} [N]$ | |
| Female | 1.92 (0.81)  n=5 | 2.68 (1.74)  n=18 | 0.507* | 4.34 (0.00)  n=1 | 4.92 (1.65)  n=5 |
| Diabetes | 1.70 (0.59)  n=3 | 2.64 (1.67)  n=20 |  | n=0 | 4.82 (1.49)  n=6 |
| MI | 4.72 (2.04)  n=5 | 1.90 (0.71)  n=18 | $\boldsymbol{5.02}\boldsymbol{10}^{\boldsymbol{-5}}$ | 7.49 n=1 | 4.29 (0.81)  n=5 |
| CADS | 2.67 (2.20)  n=8 | 2.43 (1.25)  n=15 | 0.415 | 7.49 n=1 | 4.29 (0.81)  n=5 |
| Smoker | 3.12(1.97) n=9 | 2.12 (1.23)  n=14 | 0.145 | 4.82 (1.49)  n=6 | n=0 |
| HT | 2.74 (1.74)  n=18 | 1.69 (0.21)  n=5 | 0.26 | 4.94 (1.64)  n=5 | 4.24 n=1 |
| HF | 1.54 (0.23)  n=7 | 2.94 (1.76)  n=16 | 0.086 | n=0 | 4.82 (1.49)  n=6 |
| AI | 2.09 (1.12)  n=18 | 4.02 (2.26)  n=5 | **0.017** | 3.10 n=1 | 5.17 (1.38)  n=5 |
| AS | n=0 | 2.51 (1.60)  n=23 |  | n=0 | 4.82 (1.49)  n=6 |
| Ectasia | 2.26 (1.36)  n=11 | 2.75 (1.82)  n=12 | 0.244 | 3.10 n=1 | 5.17 (1.38)  n=5 |
| AAA | 2.34 (1.32)  n=4 | 2.55 (1.68)  n=19 |  | 5.94 (2.19)  n=2 | 4.26 (0.93)  n=4 |

Supplementary Table S9- Mean, standard deviation (SD) of the peak load of the axially (-ax) and circumferentially (-circ) loaded symconCT tests on human thoracic aortic specimens from bicuspid (H-BAV) patients. The table denotes the p-values of the Linear Mixed Effect (LME) model analysis, which were computed only for a number of data (n) >4. *p-value=0.663 for the influence of smoking on H-ax-BAV. The abbreviations of the clinical parameters are listed in Table 1.

| **Clinical Parameter** | **H-ax-BAV "Yes"** | **H-ax-BAV "No"** | **H-circ-BAV "Yes"** | **H-circ-BAV "No"** |
| --- | --- | --- | --- | --- |
|  | $P_{\mathrm{peak}} [N]$ | | $P_{\mathrm{peak}} [N]$ | |
| Female | 3.62 (0.39) n=3 | 4.72 (1.74) n=8 | 4.31 n=1 | 6.05 (2.59)  n=5 |
| Diabetes | n=0 | 4.42 (1.56)  n=11 | 1.56 | 6.60 (1.43)  n=5 |
| MI | 2.94 (0.45) n=2 | 4.75 (1.53) n=9 | 5.19 (1.23)  n=2 | 6.04 (3.00)  n=4 |
| CADS | 3.84 (1.07) n=4 | 4.75 (1.76) n=7 | 5.19 (1.23)  n=2 | 6.04 (3.00)  n=4 |
| Smoker^*^ | 4.22 (2.94) n=6 | 4.66 (0.84)  n=5 | 5.38 (2.50)  n=5 | 7.65  n=1 |
| HT | 4.60 (1.73) n=7 | 4.12 (1.39) n=4 | 2.94 (1.95)  n=2 | 7.17 (0.75)  n=4 |
| HF | 5.92 (3.34) n=2 | 4.09 (0.97) n=9 | n=0 | 5.76 (2.43)  n=6 |
| AI | 3.96 (0.51) n=2 | 4.52 (1.71) n=9 | 7.54 (0.19)  n=3 | 3.98 (2.27)  n=3 |
| AS | 4.98 (1.67) n=7 | 3.45 (0.71) n=4 | 1.56 n=1 | 6.60 (1.43)  n=5 |
| Ectasia | 3.26 n=1 | 4.54 (1.59) n=10 | 6.54 (1.92)  n=3 | 4.98 (3.03)  n=3 |
| AAA | 4.59 (1.87) n=2 | 4.38 (1.61) n=9 | 4.31 n=1 | 6.05 (2.59)  n=5 |

Supplementary Table S10- Mean, standard deviation (SD) of the normalized clamp displacement at peak load, and statistical influence of clinical parameters on the normalized clamp displacement from the symconCT tests on human thoracic aortic specimens from bicuspid patients (H-BAV) and patients having tricuspid or quadricuspid aortic valves (H-noBAV). Here, axially and circumferentially loaded specimens are grouped together, given the loading direction does not influence the norm. clamp displ. The table denotes the p-values of the Linear Mixed Effect (LME) model analysis, which were computed only for a number of data (n) >4. *Log-normal data distribution. The abbreviations of the clinical parameters are listed in Table 1.

| **Clinical Parameter** | **H-BAV "Yes"** | **H-BAV "No"** | ***p*-value (LME)** | **H-noBAV "Yes"** | **H-noBAV "No"** | ***p*-value (LME)** |
| --- | --- | --- | --- | --- | --- | --- |
|  | Norm. clamp displ. | |  | Norm. clamp displ. | |  |
| Female | 0.47 (0.11) n=4 | 0.86 (0.43) n=13 |  | 0.46 (0.13) n=6 | 0.63 (0.22) n=23 | 0.076 |
| Diabetes | 0.70  n=1 | 0.77 (0.42) n=16 |  | 0.61 (0.16) | 0.59 (0.22) n=26 |  |
| MI | 0.43 (0.16) n=4 | 0.88 (0.41) n=13 | 0.084 | 0.50 (0.16) n=6 | 0.62 (0.23) n=23 | 0.234 |
| CADS | 0.52 (0.26) n=6 | 0.90 (0.42) n=11 | 0.082 | 0.52 (0.15) n=9 | 0.63 (0.24) n=20 | 0.244 |
| Smoker | 0.57 (0.25) n=11 | 1.13 (0.41) n=6 | **0.006** | 0.60 (0.29) n=15 | 0.59 (0.11) n=14 | 0.818 |
| HT | 0.55 (0.19) n=9 | 1.02 (0.46) n=8 | **0.016** | 0.57 (0.20) n=23 | 0.69 (0.28) n=6 | 0.236 |
| HF | 0.45 (0.02) n=2 | 0.81 (0.42) n=15 |  | 0.58 (0.10) n=7 | 0.60 (0.24) n=22 | 0.788 |
| AI | 1.17 (0.34) n=5 | 0.60 (0.32) n=12 | **0.015** | 0.64 (0.18) n=19 | 0.51 (0.26) n=10 | 0.106 |
| AS | 0.69 (0.34) n=8 | 0.84 (0.47) n=9 | 0.469* | n=0 | 0.60 (0.22) n=29 |  |
| Ectasia | 0.67 (0.37) n=4 | 0.80 (0.43) n=13 | 0.596 | N 0.67 (0.20) n=12 | 0.54 (0.22) n=17 | 0.131 |
| AAA | 0.74 (0.59) n=3 | 0.78 (0.39) n=14 | 0.948 | 0.63 (0.29) n=6 | 0.59 (0.20) n=23 | 0.624 |

Supplementary Table S11- Mean, standard deviation (SD) of the fracture energy (*D*), and statistical influence of clinical parameters on the fracture energy from the symconCT tests on human thoracic aortic specimens from bicuspid (H-BAV) patients and patients having tricuspid or quadricuspid aortic valves (H-noBAV). Here, axially and circumferentially loaded specimens are grouped together given the loading direction does not influence the *D* value. The table denotes the p-values of the Linear Mixed Effect (LME) model analysis, which were computed only for a number of data (n) >4. *p-value=0.002 for the influence of smoking in H-noBAV specimens. The abbreviations of the clinical parameters are listed in Table 1.

| **Clinical Parameter** | **H-BAV "Yes"** | **H-BAV "No"** | **H-noBAV "Yes"** | **H-noBAV "No"** |
| --- | --- | --- | --- | --- |
|  | $D \left[ \mathrm{kJ}m^{-2} \right]$ | | $D\left[ \mathrm{kJ}m^{-2} \right]$ | |
| Female | 1.13 n=1 | 2.09 (1.34)  n=8 | 0.65 (0.16) n=4 | 1.07 (0.80) n=12 |
| Diabetes | 0.51  n=1 | 2 .17 (1.24) n=8 | 0.56  n=1 | 0.99 (0.73) n=15 |
| MI | 0.97 n=1 | 2.11 (1.32) n=8 | 1.25 (0.69) n=4 | 0.87 (0.72) n=12 |
| CADS | 1.41 (0.40) n=3 | 2.27 (1.52) n=6 | 0.91 (0.66) n=7 | 1.01 (0.79) n=9 |
| Smoker* | 1.20 (0.71) n=4 | 2.62 (1.35) n=5 | 1.34 (0.86) n=8 | 0.60 (0.18) n=8 |
| HT | 1.42 (0.64) n=5 | 2.70 (1.64) n=4 | 1.00 (0.76) n=14 | 0.70 (0.05) n=2 |
| HF | 2.19 n=1 | 1.96 (1.38) n=8 | 0.55 (0.18) n=6 | 1.22 (0.80) n=10 |
| AI | 3.86 (1.42) n=2 | 1.45 (0.62) n=7 | 0.83 (0.73) n=10 | 1.19 (0.69) n=6 |
| AS | 1.53 (0.63) n=6 | 2.90 (1.95) n=3 | 0.97 (0.71) n=16 | n=0 |
| Ectasia | 4.86 n=1 | 1.63 (0.76) n=8 | 1.01 (0.91) n=6 | 0.94 (0.62)  n=10 |
| AAA | 2.10 n=1 | 1.97 (1.38) n=8 | 1.44 (0.71) n=3 | 0.86 (0.69) n=13 |
